# Orchard microclimate, tree water uptake and sweet cherry fruit quality under protected cropping

**DOI:** 10.3389/fpls.2022.993817

**Published:** 2022-10-18

**Authors:** Cameron Hugh Stone, Dugald C. Close, Sally A. Bound, Ross Corkrey

**Affiliations:** Tasmanian Institute of Agriculture, University of Tasmania, Hobart, TAS, Australia

**Keywords:** sap flow, relative humidity, solar radiation, wind speed, compression firmness, soluble solids, VOEN, temperature

## Abstract

Protected cropping systems (PCS) de-risk adverse climatic effects in intensive horticultural production but alter the growing environment. The objectives of this study were to investigate the effects of modern, commercial-scale PCS on sweet cherry orchard microclimate, tree water uptake and fruit quality. Sap flow sensors and weather stations were positioned at four locations under a 21 ha PCS at varying elevations (125, 114, 111, 102 m above sea level) and distances from the block boundary (105, 75, 60 or 50 m, referred to hereafter as Locations 1 to 4, respectively). Generalised additive models (GAMs) were used to predict the effect of individual climate parameters (temperature, relative humidity, solar radiation and wind speed) on tree sap flow at each of the four locations. Average and maximum temperatures and average minimum relative humidity (RH) were higher (15.9°C, 26.1°C and 49.0%) at locations with higher elevations and located further from the PCS boundary (locations 1 and 2) in contrast to locations at lower elevations and closer proximity to the PCS boundary (locations 3 and 4) (15.4°C, 24.6°C and 48.1%). Predicted sap flow was strongly correlated (r^2^ = 0.92) with time across the four locations under the PCS. GAMS modelling indicated that the hourly water uptake by trees within close proximity to the block boundary (locations 3 and 4) responded with greater intensity to increases in temperature and reductions in relative humidity, taking up on average 0.15 L h^-1^ (at temperatures >30°C) and 0.08 L h^-1^ (at RH<50%), respectively, in contrast to trees further under the PCS (locations 1 and 2) where average tree water uptake was 0.08 and 0.04 L h^-1^ at temperatures >30°C and RH<50%, respectively. Highest average predicted hourly tree sap flow was associated with high wind speeds (0.67 L h^-1^) and low relative humidity levels (0.61 L h^-1^). Fruit harvested from locations further from the PCS boundary had significantly higher dry matter content (18.2%), total soluble solids (17.8%) and compression firmness (311.3 g mm^-1^) in contrast to fruit closer to the PCS boundaries (16.1%, 15.7% and 258.3 g mm^-1^). This study provides greater understanding of the effects of PCS on microclimate and consequences for tree water uptake and fruit quality.

## Introduction

Implementation of rain covers for protected cropping of sweet cherry (*Prunus avium*) has led to an improvement in marketable yield due to a reduction in the incidence and severity of fruit cracking and disease resulting from late season rainfall events (Lang, 2013; Ruisa et al., 2017; Mika et al., 2019). However, passive protected cropping systems (PCS) can develop microclimates that have detrimental effects on fruit development and quality characteristics (Borve and Meland, 1998), with elevated average temperature and relative humidity (RH) and a reduction in solar radiation levels considered the primary causes (Meli et al., 1984; Lang, 2014). Whilst the effects of passive PCS on microclimate, fruit development and quality in an orchard setting have been well documented (Balkhoven-Baart and Groot, 2005; Lang, 2014; Wallberg and Sagredo, 2014), there is limited research on the microclimates developed under more modern self-ventilating PCS such as the Voen system (Voen, Vöhringer GmbH & Co, Berg, Germany) with a lack of information on the effects of these systems on tree water uptake.

A decline in the quality of fruit weight and firmness from trees positioned more centrally under a Voen PCS, in contrast to trees closer to the boundary, has been reported in a preliminary study (per comms. Ocean) undertaken on a relatively mild site. Stone et al. (2021) reported higher average and minimum RH levels associated with central locations that were correlated with reduced wind speeds and high temperature relative to locations closer to the PCS boundary. These authors also reported a three-fold reduction in average daily tree water uptake, relative to trees under bird-exclusion netting, for 16-year-old ‘Lapins’ grafted to ‘Colt’ in the Huon Valley, Tasmania, Australia. Given that transpiration-driven water uptake has been associated with fruit expansion (Morandi et al., 2007) and high light and rates of photosynthesis are associated with fruit yield (Wűnsche et al., 1996), firmness and soluble sugars (Zhang et al., 2018) and that wind exposure has a significant effect on sap flow in sweet cherry (Stone et al., 2021) we expected to find a gradient where temperature and RH elevated with distance from the boundary of the PCS and we hypothesised that this gradient would be negatively associated with gradients in fruit quality. Therefore the aim of this study was to investigate temperature, RH, solar radiation, and wind speed across an altitude and proximity to PCS boundary gradient under a large (21 ha) self-ventilating PCS. Using generalised additive models (GAM), we were able to investigate the effects of climate variables on tree sap flow across this gradient and potential impacts on fruit quality.

## Materials and methods

### Site characteristics

Field research was undertaken during the 2020-21 growing season (October-March) on a commercially managed sweet cherry orchard at Rosegarland in the Derwent Valley, Tasmania, Australia (42° 41’ S, 146° 56’ E).

The trial was established in a 21-ha block on a south-western slope (11%) with Voen rain covers. Trees were 14-year-old ‘Staccato’ on ‘Colt’ rootstock trained to a Spanish bush system planted at 2 m within and 5 m between row spacings with row lengths of 105 m. Soil profile comprised of a grey Kandosol with a heavy clay topsoil and the presence of course rock fragments in the upper 600 mm (Musk and De Rose, 2000). The orchard was subjected to standard industry dripper irrigation, fertiliser and pest and disease control regimes.

### Trial design

Four locations with different above sea level (asl) elevations and distances from the PCS boundary (DFB) were chosen as described in **Table 1**. Two trees (within a four-tree span) were randomly selected and tagged at each location (eight trees in total). Trunk cross sectional areas were calculated from the measurements of trunk circumferences 10 cm above the graft union for each tree. Two representative branches were selected and tagged on each tree for flower and fruit set counts, and sampling for subsequent harvest and analysis of fruit quality characteristics.

**Table 1:** Elevation and distance from the boundary of four study locations under a 21-ha rain covered protected cropping system.

Location	Elevation above sea level(m)	Distance from boundary(m)
1	125	105
2	114	75
3	111	60
4	102	50

### Sap flow

A single set of heat-pulse probes (Tranzflo NZ Ltd, Palmerston North, New Zealand) was inserted into the trunk of two trees at each location under the PCS (eight trees in total). Each set of heat-pulse probes was made from two 19-g stainless steel hypodermic tubes, with four thermocouples at depths of 5, 15, 25 and 40 mm insulated with a Teflon sheath. Three vertically aligned holes were drilled into the northern side of the trunks of each tree using a steel drill guide and a 1.95 mm drill bit approximately 10 cm above the graft unions. A corer was used to remove the bark surrounding the drilled holes, ensuring the 5 mm thermocouple was positioned as close to the cambium as possible with standard spacing of 5 mm upstream and 10 mm downstream from the heater probe (Doley and Grieve, 1966). Probes were insulated from direct solar radiation by wrapping aluminium foil around the trunk of the tree. The standard compensation heat pulse method (CHPM) (Green and Clothier, 1988; Green et al., 2018) was used to calculate daily transpiration rates from the sap flux velocities. Data loggers (model CR1000, Campbell Scientific, Logan, USA) measured the time taken to achieve thermal equilibrium between the sensors located 10 mm above and 5 mm below the heater probe following the application of a 2.5 s heat pulse into the conducting wood area of the tree. Sap flow velocity multiplied by the cross-sectional area of the conducting wood gives the volume flow per unit time. The heat pulse generated by each probe was regulated according to Green et al. (2018) to ensure delivery of 50 J of energy each time the probes were fired. Data was collected at thirty-minute intervals between mid-October 2020 and late March 2021. Conducting wood area was calculated after taking core samples from representative trees. Daily sap flow was calculated using the approach outlined by Green et al., 2003; Green et al., 2009), with calculations accounting for the wounding effect. A wound diameter of 2.8 mm was determined from the 1.98 mm diameter drill holes using the theoretical calibrations described by Swanson and Whitfield (1981).

The data were obtained from two trees per location, which is the minimum possible number from which statistically valid estimates could be obtained. They were chosen by random selection and therefore we do not have any reason to question their representativeness. Since we lack data on trees per location that could have been sampled, but were not, it is not possible to estimate the degree to which estimates obtained from the two sampled trees were representative of the others. Equally, we have no reason to think them unrepresentative.

### Climate

Climate data was collected using Hobo U30-NRC weather stations (Onset, USA) at each of the four locations where sap flow measurements were recorded. An additional weather station was located outside the PCS block. Weather stations at each location measured air temperature (°C), RH (%), global solar radiation (MJ m^-2^), wind speed (m s^-1^) and soil moisture (m^-3^) (at 50, 100 and 200 mm depth) at thirty-minute intervals. Data was collected between October 2020 and April 2021. Estimations of respective hourly and daily vapour pressure deficit (VPD) and crop factors were used to calculate the estimated reference crop evapotranspiration (ETo) for each location (Allen et al., 1998).

### Fruit crop load and quality

Prior to harvest crop load measurements were taken for each tree. The trunk cross sectional area of each tree was calculated from trunk circumferences measured approximately 10cm above the graft union. Fruit counts were taken on the tagged branches in each tree. Fruit was harvested from the tagged branches 96 days after full bloom (DAFB) in late January 2021 at standard commercial harvest time. All fruit from tagged branches were picked irrespective of quality and placed into sealed bags. Bulk field weight measurements were taken for each branch using electronic scales (Jastek) prior to being transported and fruit was stored at 4°C prior to grading within 24 hours.

Total fruit counts for each branch were recorded and fruit was then graded into first class, second class and reject (cracked, rotten or damaged) based on size and colour determined by the Australian cherry colour guide (Cherry Growers Australia). A sample of 30 first class fruit was randomly selected from each tree for assessment of fruit quality parameters (diameter, weight, compression firmness, skin colour, stem pull force, total soluble solids (TSS) content and dry matter content (DMC)).

Cherry colour was measured using a Konica Minolta, CR-400 Chroma Meter (Konica Minolta Sensing, Inc., Osaka, Japan), with two measurements per fruit, one on each side of the cherry. Results were expressed in the CIELAB or L*a*b* format (a colour space defined by the International Commission on Illumination) with only L* reported in this study. Fruit diameter was measured across the widest points (cheeks) of the individual cherries to one decimal place using digital vernier calipers (Sidchrome, SCMT26226). Weight with stem attached was measured using Mettler Toledo scientific balance scales to one decimal place. Compression firmness for individual fruit was measured using a Firmtech 2 (Bioworks Inc., Stillwater, Okla., USA). Cherries were placed with their cheek (widest point) on a horizontal axis rather than vertical due to the large fruit size. Stem pull force (g) was measured using a stand mounted Mark-10 Series 5 Force gauge (Mark-10, USA). Dry matter content was measured using half the fruit from each sample; fruit were de-pipped and dried at 68°C for a week. The remaining fifteen fruit were juiced to measure fruit TSS (°Brix) with a PAL-1 digital handheld refractometer (Atago, Japan).

### Data analysis

The sap flow data was analysed in three stages. The first stage fitted a nonlinear repeated measures model that used time alone as the sole predictor for sap flow. The distribution of the data was approximated using a bell-shaped curve in the form:


$$Sap\;Flow\;\left(SF\right)=B\;x\exp\left(-\frac{{\left(Halfhour-\;M\right)}^{2}}{S}\right)$$

Equation 1

Abbreviations for each term are explained in **Table 2**. Parameter ‘B’ controls the height; ‘M’ the time-position of the peak; and ‘S’ the width of the peak in the bell-shape curve. The time-only model allowed for random variation in ‘B’ between days to be incorporated in the estimation by means of a random effect per day. The time only model was fitted using PROC NLMIXED in SAS version 9.4.

**Table 2:** Abbreviations for terms used in equations 1 and 2.

Term	Interpretation
SF	sap flow
M	mean half hour when the sap flow is maximum
S	variability for the time when sap flow was maximum
B	mean estimate for the maximum sap flow
sb	variance for the random tree effect
S2	variance for the prediction
BT0	regression intercept
BT1	linear effect for temperature
BT2	quadratic effect for temperature
C1	linear effect for solar
C2	quadratic effect for solar
R1	constant effect for RH ≤ 60
R2	linear effect for RH>60
Ws1	linear effect for wind speed
Ws2	quadratic effect for wind speed

Once a fit had been obtained, (i.e., predictions minus observed data) residuals from the time-only model were examined graphically in R by plotting the residuals against time (thirty-minute intervals). This was undertaken to assess if the model fitted all times equally well, or if there were times when it fitted less well.

Generalised additive models (GAM) were used to further examine the residuals that did not fit time well. The GAM models were fitted to the residuals of the time-only model using temperature, solar radiation, RH and wind speed as predictors. This allowed the effects of temperature, solar radiation, RH and wind speed on ‘B’ to be visually assessed within a non-parametric framework after having already allowed for the bell-shaped time-variation. The GAMs were intended to serve an exploratory purpose rather than an inferential one. They were used to identify if, once the effect of time had been removed, the dependence of sap flow on the predictors could be modelled as polynomials or some other function. The GAM models were fitted using the ‘mgcv’ package in R version 3.6.

The visual trends for each variable thus obtained were complex. On examination, quadratic trends for all variables except for RH were identified while RH had a segmented model: two linear regressions with a breakpoint at RH=60% (**Appendix A1**).

Using the functional form of the relationship identified from the GAMs, the nonlinear model was rerun in SAS (PROC NLMIXED in SAS version 9.4) with time, as before, but with the addition of functions of temperature, solar radiation, RH and wind speed as suggested by visual examination of the GAM fits.

Below is the nonlinear model including assumed relationships from the GAM models with temperature, solar radiation, RH and wind speed, as well as time. For RH a segmented model was fitted with a linear slope for RH ≤ 60%, and a slightly different slope for RH > 60%.


SF=BT0+(linear and quadratic terms)+ {R1×RH,if RH≤60R2×RH,if RH>60

Equation 2

If the R2 term was zero then there was no change in slope at RH = 60. Temperature was divided by 100 in the fitted model to avoid numeric overflow.

An alternative graphical method used smoothed median trends of the residuals against the four covariates. The median trends were similar to the trends shown in the GAM plots. Modelled data provided a visual representation of how the diel trend of sap flow varies in response to the predictors of temperature, RH, solar and wind speed one at a time rather than specifically providing a prediction against the four variables

Analysis of the fruit quality data was completed using RStudio (v.1.2.5019) with ANOVA and Tukey comparison of fruit means calculated for each location.

## Results

### Microclimate

Average maximum temperatures were significantly lower (1.8°C) at location four and in the open block compared to locations one, two and three (**Table 3**). Average daily maximum temperatures and night-time minimum temperatures were significantly (p ≤ 0.001) higher for location two (higher asl elevation, furthest from PCS boundary) in contrast to location four (lower asl elevation, closer to PCS boundary). There was no significant difference between other locations. Average daily RH and average minimum RH were similar across all locations under the PCS yet were significantly higher (7.5% and 9.5% respectively) relative to the adjacent open block. Average total daily solar radiation was reduced by 38.7% and average wind speeds were reduced six-fold under the PCS. Maximum VPD values reduced with the declining gradient of the block. Average ETo under the covers (2.3 to 2.5 mm) was lower in contrast to measurements in the adjacent open block (3.9 mm).

**Table 3:** Average daily climate data and sap flow over the season between October 17, 2020, and March 31, 2021, for location 1 (125 m asl, 105 m DFB), 2 (114 m asl, 75 m DFB), 3 (111 m asl, 60 m DFB), 4 (102 m asl, 50 m DFB) and in an adjacent open block.

a.	Location	Temp	Max T	Min T	RH	Min RH (%)	Solar radiation (MJ m^-2^)
		(°C)	(°C)	(°C)	(%)		
	1	15.9	25.7 b	8.5	74.0 b	47.6 b	11.5 a
	2	15.9	26.4 b	8.1	75.6 b	48.6 b	12.4 b
	3	15.7	25.5 b	8.2	75.8 b	49.0 b	11.6 a
	4	15.1	23.7 a	8.1	74.6 b	48.9 b	12.5 b
	open	15.1	22.4 a	8.4	69.8 a	44.3 a	19.4 c
**b.**	**Location**	**Wind speed (m s^-1^)**	**Ave Soil moisture**	**Max VPD (kPa)**	**ETo**	**GDD**	**Sap flow (L d^-1^)**
			**(m^3^/m^3^)**		**(mm d^-1^)**		
	1	0.36 a	0.16 b	1.81 b	2.3 a	1197 b	5.8 a
	2	0.53 b	0.18 c	1.84 b	2.5 a	1222 b	6.0 a
	3	0.56 b	0.11 a	1.72 ab	2.3 a	1176 b	7.1 b
	4	0.40 a	0.20 d	1.56 a	2.4 a	1094 a	5.7 a
	open	2.97 c	–	1.62 ab	3.9 b	1030 a	–

Letters within columns illustrate significant difference between locations (p ≤ 0.05). asl, above sea level; DFB, distance from boundary; Temp, temperature; Max T, maximum temperature; Min T, minimum temperature; RH, relative humidity; Min RH, minimum relative humidity; Max VPD, maximum vapor pressure deficit; ETo, evapotranspiration; GDD, growing degree days (Hochmaier, 2014).

### Sap flow

Average daily tree sap flows across the trial site were similar with only location three being significantly different (7.1 L day^-1^) in contrast to locations one, two and four (5.8, 6.0 and 5.7 L day^-1^ respectively). Overall, an average of 6.2 L day^-1^ was measured across all locations for the season (October to March).

Daily average tree water uptake significantly and positively correlated with increases in solar radiation (r^2^ = 0.73, p =< 0.001), soil moisture content (r^2^ = 0.41, p =< 0.001) and air temperature (r^2^ = 0.18, p =< 0.001), while a significant negative correlation was found between average daily sap flow and increasing average RH (r^2^ = 0.45, p =< 0.001) (raw data; not shown). Although significant (p< 0.001), the correlation between average daily tree sap flow with increasing wind speed was not strong (r^2^ = 0.03).

**Figure 1:**
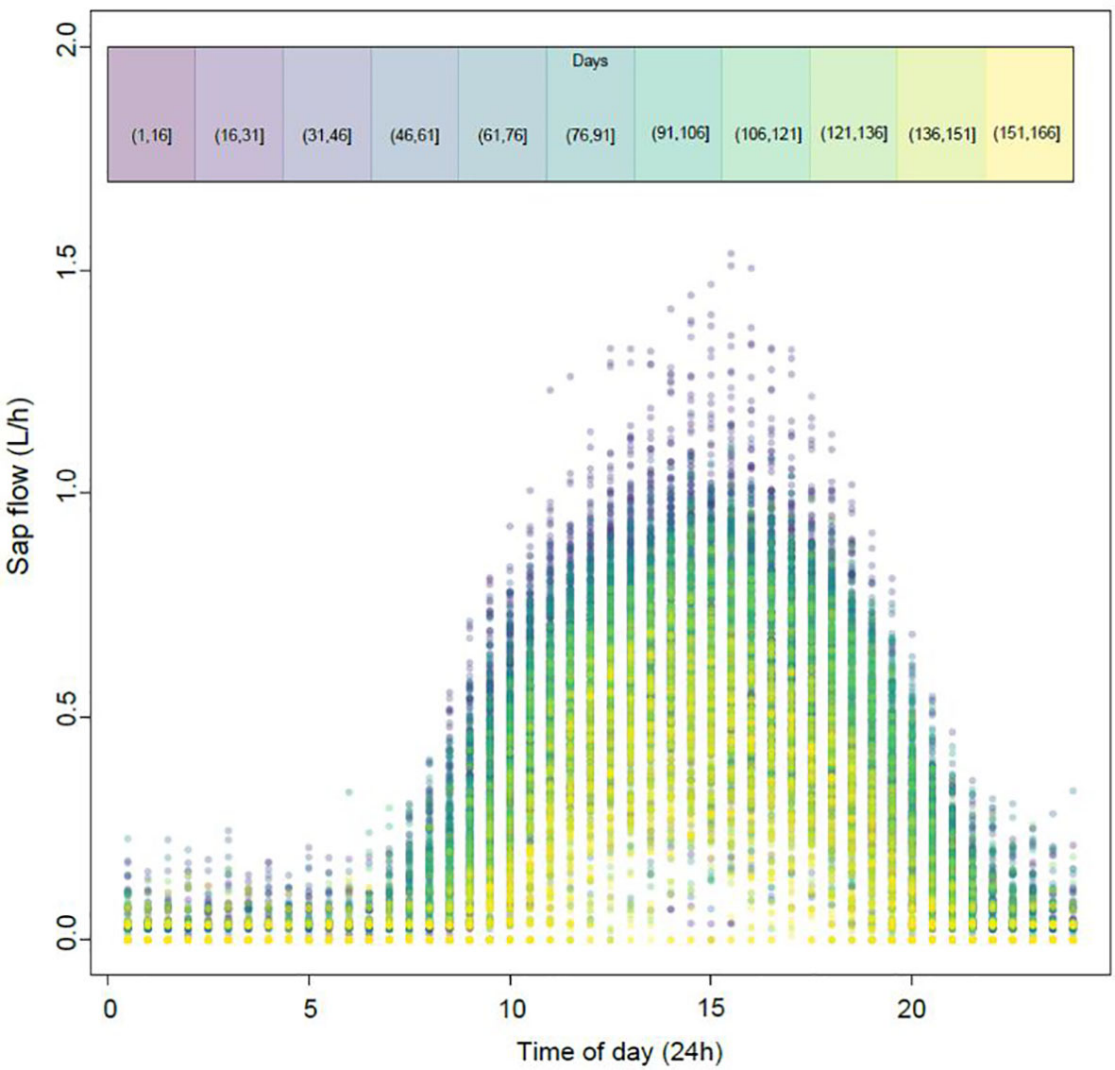
Pooled observations of daily sap flow data recorded every 30-minutes at four different locations under a Voen protected cropping system. Day 1 was October 17^th^ 2020. Dot colour represents the different time periods throughout the season as highlighted in the colour chart at the top of the figure (eg. Purple represents days 1-16, yellow represents days 151-166).

Daily variation of sap flow pooled from all four locations followed a bell-shaped curve peaking at approximately 3 pm (**Figure 1**). Variation within the season was considerable with higher sap flows of approximately 7.1 L day^-1^ measured between days 1-91 (mid-October to mid-January) relative to sap flows later in the season between days 91-166 (late January to late March) where average daily sap flows were 4.2 L.

**Figure 2:**
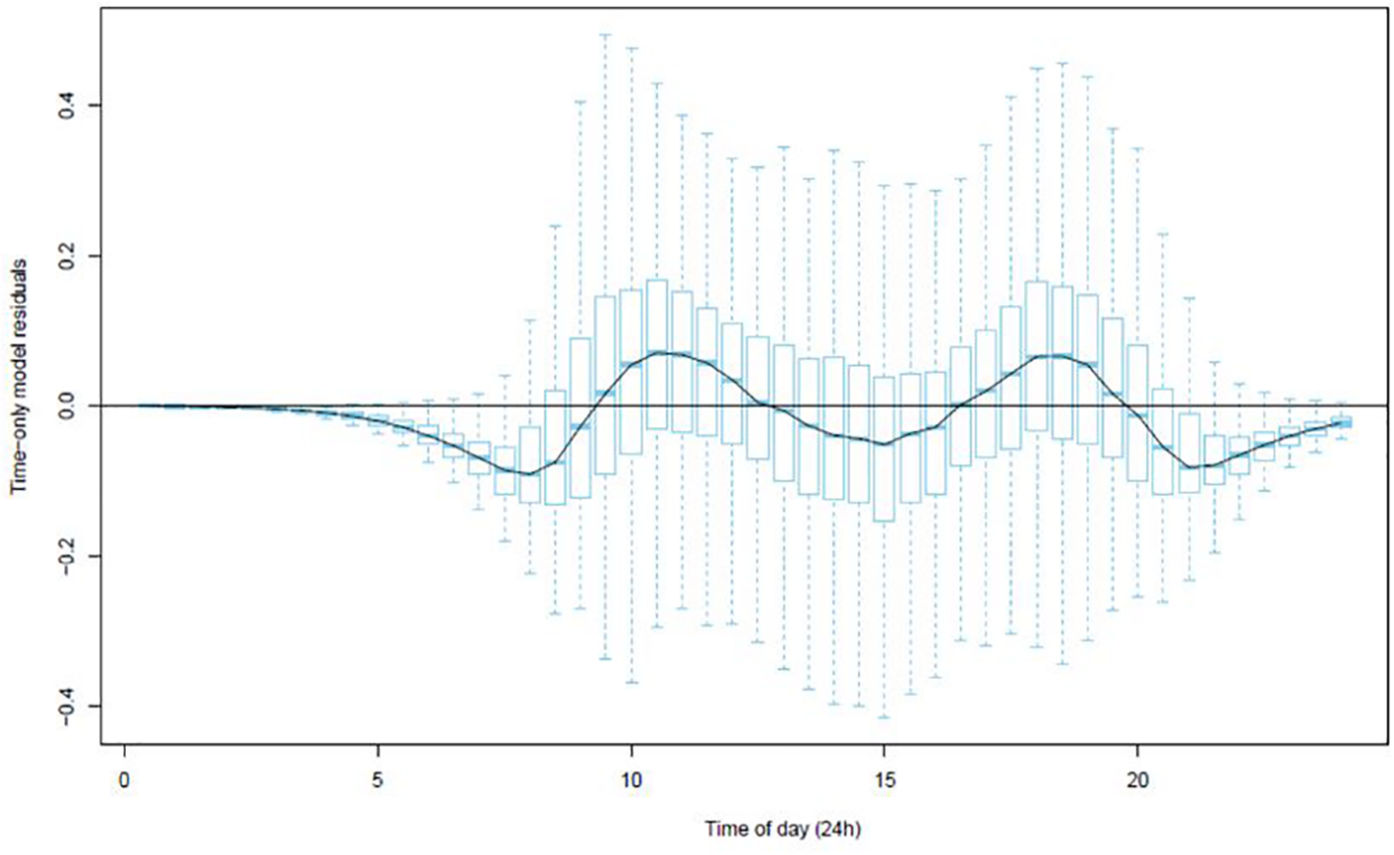
Boxplot of the residuals from the time-only model (Equation 1) at each 30-minute interval.

The residuals from modelling sap flow against time (**Figure 2**) indicated that whilst the model explained the data well there were negative departures in the early morning and late evening where the residuals fell below zero. This suggested that there were some inadequacies with the time-only model of predicted sap flows.

To further improve the model the effects of temperature, solar radiation, RH and windspeed were investigated and added (Equation 2).

### GAM models of sap flow

**Figure 3:**
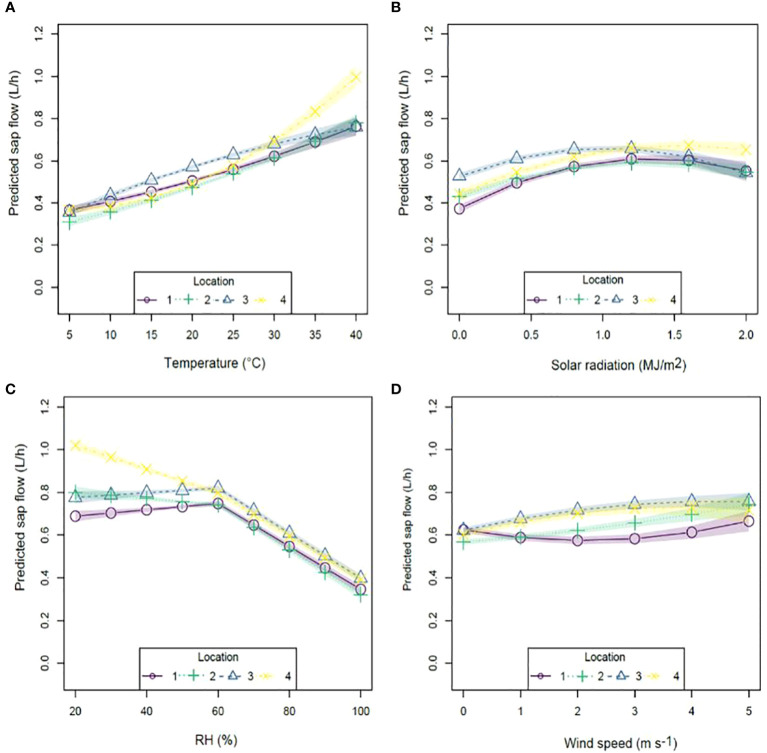
Modeled sap flow fit for **(A)** temperature; **(B)** solar radiation; **(C)** relative humidity and **(D)** wind speed using the nonlinear model (Equation 2) by condition at 12 pm. Common scaling.

The addition of climatic variables to equation 2 enabled a comparison of the individual effect of different variables on hourly tree sap flow. Investigation of each predictor, while keeping all others constant, revealed effects of altered rate of sap flow at each location (**Figure 3**) that were similar in pattern to that observed in the raw data, but more powerful due to the capacity of the model to eliminate the confounding effects of other parameters. Predicted sap flow increased with increasing temperature at locations one and two, with a more definite increase at location four at temperatures above 25°C (**Figure 3A**). Greatest variation in sap flow occurred at location four with an increase of approximately 0.62 L between 5 – 40°C in contrast to an increase of approximately 0.38 L at locations one two and three within the same temperature range.

Predicted sap flows had a parabolic response to solar radiation (**Figure 3B**) for all locations with a peak at approximately 1.2 MJ m^-2^ at locations one, two and three, while location four peaked at approximately 1.6 MJ m^-2^. The greatest variation in predicted sap flow occurred at location one, with an increase of approximately 0.24 L between 0 – 1.2 MJ m^-2^.

Predicted sap flow increased slightly between 20-60% RH (**Figure 3C**) at locations one and three (increase of approximately 0.03 L) however this was not significant (p ≥ 0.05) in contrast to locations two and four where predicted sap flow significantly declined by approximately 0.08 L and 0.21 L, respectively (**Appendix A2**). In contrast, predicted sap flows at all locations declined significantly by approximately 0.4 L at similar intensities between 60-100% RH. Wind speed (**Figure 3D**) had varying effects on predicted sap flow with locations three and four responding in a parabolic fashion with a peak at 4 m s^-1^ resulting in sap flows of approximately 0.76 L and 0.72 L respectively. In contrast, location two increased exponentially peaking at approximately 0.74 L at wind speeds of 5 m s^-1^, while location one had a U-shaped response to increasing wind speeds with a peak at 0.66 L at 5 m s^-1^.

Of all climatic variables measured, highest predicted sap flow measurements per hour were associated with higher temperatures and lower RHs.

### Fruit quality

**Figure 4:**
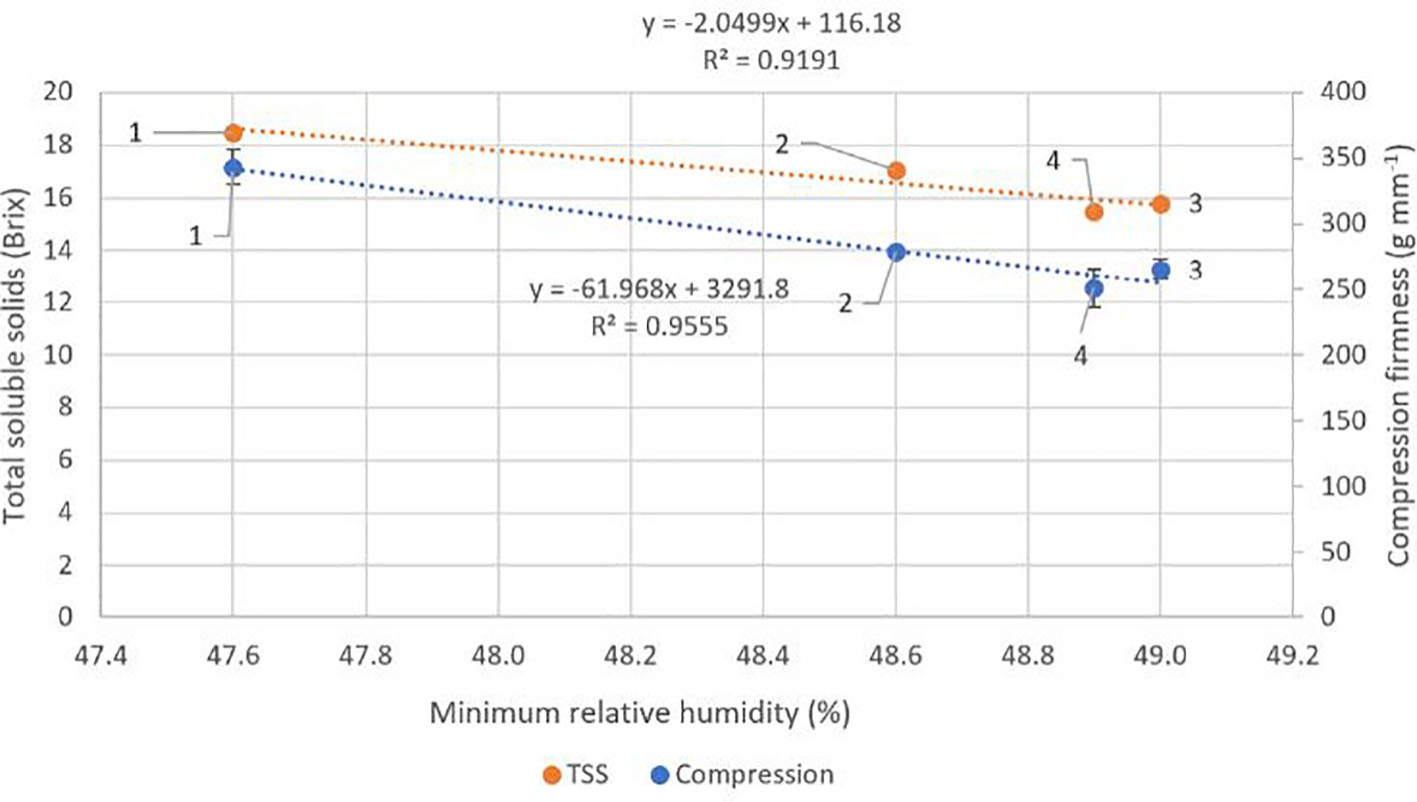
Effect of mean minimum relative humidity at each of the four locations across an altitude/distance from PCS boundary gradient on mean fruit compression firmness and mean total soluble solids (TSS). At each location n = 60, 30 fruits per tree. Bars represent one standard error for each location. PCS, protected cropping system.

**Figure 5:**
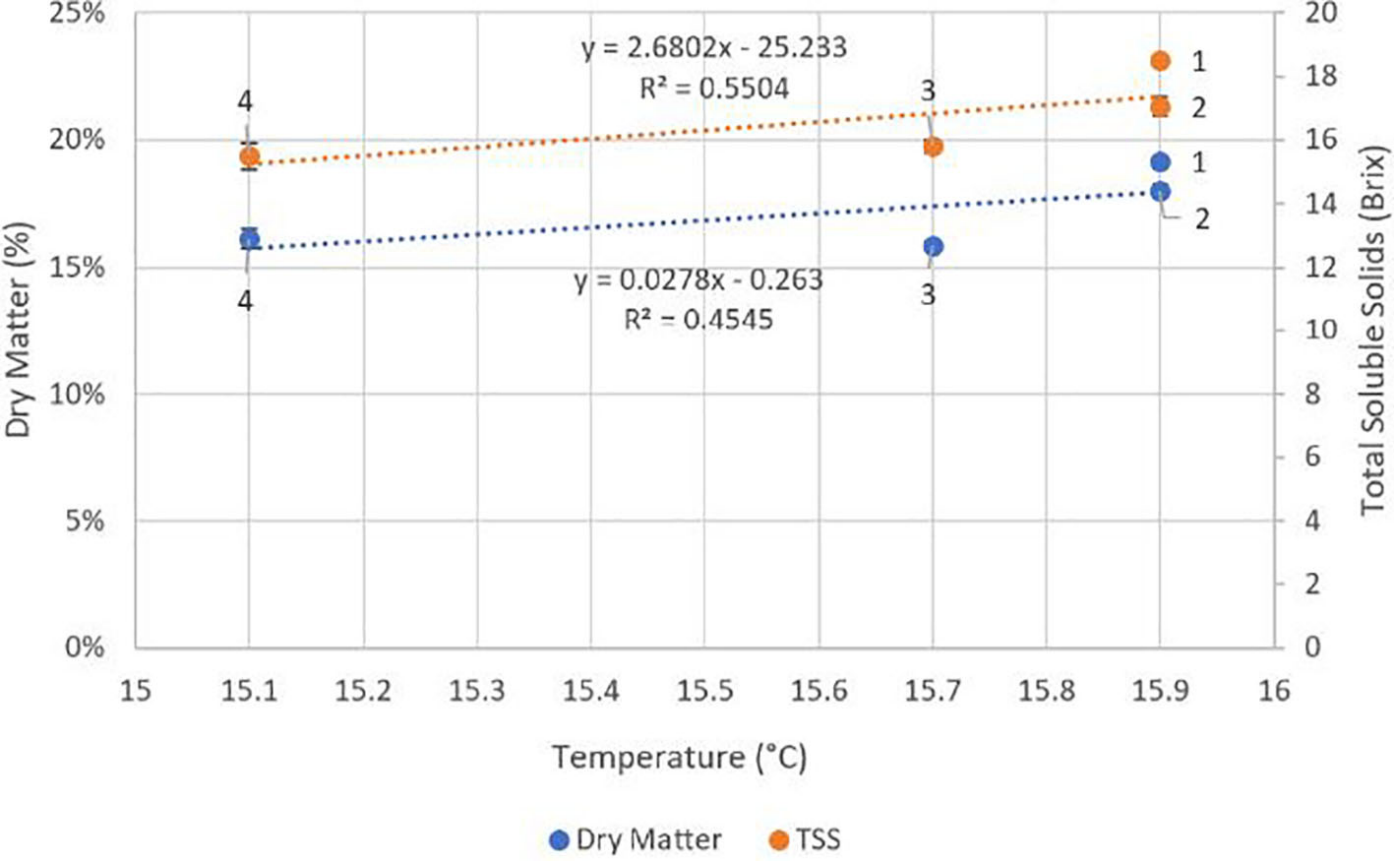
Effect of average temperature at each of the four locations across an altitude/distance from PCS boundary gradient on fruit dry matter content and total soluble solids (TSS). At each location n = 60, 30 fruits per tree. Bars represent one standard error for each location. PCS, protected cropping system.

There were no significant differences (p ≥ 0.05) between locations for crop load, fruit weight, diameter, stem pull force or colour (**Table 4**). Fruit DMC was significantly higher at locations one and two (p ≤ 0.05) compared to locations three and four. TSS and fruit compression firmness were significantly higher (p ≤ 0.05) at location one than at locations three and four. There were significant negative correlations between fruit compression firmness (r^2^ = 0.96, p = 0.02) and TSS (r^2^ = 0.92, p = 0.04) with average minimum RH across the four locations (**Figure 4**). There were trends towards correlations between average temperature and fruit DMC and TSS with r^2^ values of 0.55 and 0.45 respectively, however these results were not significant (p ≥ 0.05) (**Figure 5**).

**Table 4:** Fruit quality at harvest (96 days after full bloom).

Location	Crop load (# fruit cm^-2^ TCSA)	Wt (g)	Diam (mm)	Stem pull force (g)	DMC (%)	TSS (Brix)	Colour (L*)	Compression firmness(g mm^-1^)
1	9.2 ± 2.3	16.0 ± 0.1	32.7 ± 0.1	1182 ± 25.9	19.2 ± 0.1b	18.5 ± 0.2 b	30.4 ± 0.1	343.2 ± 6.7 b
2	14.0 ± 2.4	15.4 ± 0.2	31.6 ± 0.2	1168 ± 25	18.0 ± 0.6 b	17.1 ± 0.6 ab	30.8 ± 0.2	279.3 ± 4.5 ab
3	12.4 ± 2.0	15.0 ± 0.2	31.2 ± 0.2	947 ± 26.8	15.9 ± 0.3 a	15.8 ± 0.3 a	34.2 ± 0.4	265.8 ± 6.4 ab
4	7.2 ± 0.5	15.6 ± 0.2	31.9 ± 0.2	970 ± 29.1	16.2 ± 0.6 a	15.5 ± 0.6 a	34.3 ± 0.5	251.1 ± 5.8 a

Within each column different letters represent significant differences (p ≤ 0.05) in the means as calculated by Tukeys comparison of means test. At each location n = 60, 30 fruits per tree. Error values represent one standard error for each location for each fruit quality characteristic. TCSA, trunk cross-sectional area; Wt, weight; Diam, diameter; Skin, skin puncture force; DMC, dry matter content; TSS, total soluble solids content; L*, colour (light/dark).

## Discussion

### Microclimate at different elevations/distance from PCS boundary

The higher daily average and maximum temperatures observed at locations one (125 m asl, 105 m DFB) and two (114 m asl, 70 m DFB) in contrast to locations three (111 m asl, 60 m DFB) and four (102 m asl, 50 m DFB) under the PCS, although not large, are likely to have been influenced by the difference in elevation and distance to the PCS boundary between the locations. We suggest that a lack of wind to displace the external PCS vents combined with the rise of warm air was the cause of the higher temperatures at the higher elevations and greater distances from PCS boundary given that the study site was situated on a sloping block (11%). Location one, located at the highest elevation and furthest from the PCS boundary, had the lowest average minimum RH (47.6%) as well as the lowest average wind speeds (0.36 m s^-1^), whilst in contrast location three, located at a lower elevation closer to the PCS boundary, had the highest average minimum RH (49%) and highest average wind speeds (0.56 m s^-1^). These results are counter intuitive to the generality of wind decreasing RH levels (Holmes and Farrell, 1993). A possible explanation for this could be structural and temporal, with the vents of the PCS occasionally sticking open, whilst the relatively high minimum RH was measured at different times to this occurrence. A stuck open vent in the cover would allow the direct removal of humid air at that location.

We expected to observe higher RH along the western edge of the PCS in contrast to further from the PCS boundary as a result of high moisture levels in the prevailing winds due to La Nina conditions that were present during the trial. This however was not evident and indicates that the effect of a rise in elevation under a large continuous PCS may have had more overall impact on average temperature and RH than proximity to block boundaries in this season.

### Estimated sap flow over time

The time only model (Equation 1) predicted sap flow under the PCS well with r^2^ values of 0.91, 0.94, 0.91, 0.93 for locations one to four respectively. Similar daily sap flow prediction against time under a vented PCS with an r^2^ fit of 0.88 was reported by Stone et al. (2021).

Correlation of observational data indicated an increase in average daily tree water uptake under conditions of high solar radiation, warm temperatures, low RH, and the presence of wind. Further, the significant positive correlation between soil moisture content and tree sap flow suggests that daily tree water uptake may have been limited at times by soil moisture content, contributing to variation seen in the other correlations between tree sap flow and climate parameters. However, given that the average sap flow across all trees of 6.2 L day^-1^ was above that measured in similar aged trees on the same rootstock under a vented PCS by Stone et al. (2021) (4 L day^-1^) in a cooler growing region during a mild season, water limitation is not considered to be a major factor in this trial.

### Estimated responses of tree water uptake to climate variables

The GAM approach enabled assessment of sap flow response to non-temporal predictors. This approach enabled the model to be constructed based on the behaviour of the data rather than on theoretical assumptions and allowed us to investigate the effects of individual climate parameters.

#### Temperature and RH

The exponential increase in predicted sap flow at locations one, two and four with increasing temperature up to 40°C, and the predicted average tree water uptake of 0.57 L h^-1^ measured in this study contrasts with the findings of Stone et al. (2021) who reported average tree water uptake predictions of 0.48 L h^-1^ between 5 - 40°C. The 0.9 L h^-1^ higher sap flow measured in this study may be due to various factors such as cultivars, graft union compatibility of the trees, soil type and depth and therefore soil water holding capacities. Intuitively, tree sap flow was highest on sunny days under conditions of high VPD and lowest on cooler, overcast days with low VPD. Alarcon et al. (2000) reported similar hourly tree sap flows of approximately 0.6 L h^-1^ in young apricot trees which is a remarkable similarity given trees were only three years old and grown in pots within a greenhouse in contrast to our study in the field of mature trees. The GAM approach indicated that when soil moisture was not limiting, maximum hourly water uptake by the trees was not reached even when temperatures reached upwards of 40°C when all other climate predictors are held constant.

A distinct and significant increase in predicted sap flow at temperatures higher than 25°C was observed for location four. This may have been due to the tree canopies at this location intercepting direct unobstructed sunlight through open/damaged vents in the PCS (reported by the orchard manager) resulting in greater canopy transpiration rates and photosynthesis. This is consistent with the solar radiation results at location four, that peaked at approximately 1.6 MJ m^-2^, well above that of the other locations which peaked at around 1.2 MJ m^-2^.

A critical threshold of 60% RH was found above which sap flows dramatically reduced at a similar rate up until 100% RH for all locations. Alarcon et al. (2000) reported a close correlation (*r^2^* = 0.89) between estimated tree transpiration measured gravimetrically and average sap flow using the heat pulse technique in potted apricot trees under PCS. Further, Winkler et al. (2020) reported reductions in transpiration at high (91.6%), in contrast to low (26.1%), RH levels. Taken together these findings indicate a strong coupling, driven by transpiration, between sap flow and the effects of RH. In this study, locations closer to the western edge of the PCS used 0.22 L more water at RH levels between RH 20-100% (0.57 L), in contrast to locations furthest from the western edge (0.35 L). This suggests that the environment further from the boundary of a PCS is more stable (consistent with wind data), resulting in reduced predicted tree water uptake in response to RH levels between 20-60% in contrast to locations in closer proximity to the block boundaries. A substantial decline in predicted sap flow (0.21 L) in response to RH levels rising from 20 to 60% was predicted at location four. This location, close to the block boundary and in close proximity to prevailing winds, would be exposed to a greater range of weather conditions (wind speeds, temperature and RH) in contrast to trees further from the boundary where climatic variables were buffered. This is of particular interest regarding irrigation and fertigation practices as knowledge that trees within close proximity to the block boundaries have higher tree water uptake will inform more frequent irrigation in order to avoid a negative impact on tree health and fruit production. Overall, maximum variation in predicted sap flow over the four locations was attributed to temperature and RH (relative to the other parameters – see next sections) with an approximate variation of 0.5 L day^-1^ between 5 – 40°C or 20 – 100% RH.

#### Solar radiation

Predicted sap flows in response to solar radiation were consistent with those reported by Stone et al. (2021), with a similar parabolic response to increasing solar radiation with peaks at approximately 1.2 MJ m^-2^ h^-1^ for locations one, two and three, while location four, closer to the PCS boundary, peaked at 1.6 MJ m^-2^ h^-1^. However, variation in total predicted sap flow reported by Stone et al. (2021) was greater (0.3 L hr^-1^) between 0-1.2 MJ m^-2^ h^-1^ in contrast to the average measurement of 0.19 L hr^-1^ across the four locations in this study. Variation between the two studies may be a result of cultivar and graft union differences between the two studied sites as previously discussed, as well as the different growing region, season and block aspect, with average total daily solar radiation of 12.9 MJ m^-2^ reported by Stone et al. (2021) being higher than the 11.9 MJ m^-2^ measured in this study. The parabolic trend indicates that a critical cut-off threshold was reached at each location regarding increasing light intensity and predicted sap flow when all other climate parameters were held constant. Zeppel et al. (2004) reported reduced sap flow in afternoons, relative to mornings, at similar VPDs in native *Eucalyptus crebra* F. Muell that could have been influenced by elevated temperatures, light intensities and/or reduced soil water availability. Our study provides clarity on the influence of light, as distinct from temperature, in sweet cherry given that predicted sap flows reduced with light intensities above 1.2 - 1.6 MJ m^-2^ h^-1^, yet they increased exponentially with rising temperatures. This may indicate that in an irrigated sweet cherry orchard, tree sap flow (and by extension transpiration controlled by stomatal closure) is restricted by reduced stomatal conductance in response to light as a cue, and not temperature. This theory requires further research.

#### Wind

Average daily wind speeds of 0.36 and 0.40 m s^-1^ were significantly lower at locations one and four in contrast to locations two (0.53 m s^-1^) and three (0.56 m s^-1^). Higher average daily wind speeds at locations two and three may have caused favourable conditions for higher rates of transpiration, resulting in an increase of average daily sap flow at these locations (Alarcon et al., 2000). Juhász et al. (2013) reported an increase in sap flow in sweet cherry under hot (26.9°C) and windy (2.5 m s^-1^) day time conditions, while Green et al. (2003) reported increases in nocturnal sap flow in apples on warm, windy nights when VPD remained elevated. In this study sap flow responded in a parabolic fashion to increasing wind speeds at locations three and four in contrast to an exponential response at location two and a U-shaped response at location one. The U-shaped trend at location one is probably due to a lack of data underpinning the model, hence should not be given weight. It is expected that an increase in wind speed results in a reduction in the thickness of the boundary layer surrounding the leaves, subsequently increasing transpiration and therefore sap flows (Lei et al., 2010). This is consistent with the modelled results of trees closer to the boundary than location one. Where the predicted sap flow response was parabolic a maximum occurred at wind speeds of 4 m s^-1^, a two-fold increase to that reported by Stone et al. (2021) of approximately 2 m s^-1^. These results are noticeably lower than reported at wind speeds of 8 m s^-1^ for a single *Pinus macrocarpa* in a wind tunnel experiment (Chu et al. (2009). Larger canopies as a result of orchard management practices as well as the development of thicker leaves (the latter representing acclimation) in response to relatively high wind speeds throughout the season (0.46 m s^-1^) may be factors in this discrepancy, however this theory warrants further research. Overall, increases in wind speed did not greatly alter predicted tree water uptake with the greatest variation between 0 and 5 ms^-1^ being approximately 0.18 L hr^-1^ at location two. However, wind speeds had the greatest influence on average predicted tree water uptake during the season when all other climatic variables were held constant, with an average of 0.67 L hr^-1^, in contrast to RH (0.61 L hr^-1^), temperature (0.57 L hr^-1^) and solar radiation (0.54 L hr^-1^). To our knowledge this is the first study focussing on the individual climatic predictor of wind speed and the effect it has on water uptake in fruit trees when all other variables are held constant in a commercial orchard environment.

### Fruit quality

#### Fruit compression firmness

In contrast to our hypothesis fruit compression firmness at location one was significantly higher (343.2 g mm^-1^) than at location four (251.1 g mm^-1^) despite being situated furthest from the PCS boundary and associated with higher average and maximum temperatures. These results contradict the findings of Ocean (pers comm) and were unexpected as it was assumed that higher temperatures could potentially compromise fruit quality characteristics as reported by Lang (2013) and Lang (2014) who found significant fruit softening on the tree when exposed to higher temperatures (+10−15°C) developed under high tunnel (HT) PCS. Both Ogden and Van Iersel (2009) and Blanco et al. (2019) reported maximum temperature increases of between 3-15°C under HT PCS compared with outdoor conditions; this increase in maximum temperatures is thought to have negatively affected fruit firmness. In this study the average maximum temperatures for all location under the PCS was 25.3°C in contrast to 22.4°C outside the PCS. The highest average maximum temperatures measured at locations one and two (25.7 and 26.4°C) although significant were only 2.7°C higher in comparison to location four (23.7°C) which had the coolest average maximum temperature. It is believed that in the presence of wind the PCS vents allowed excessive heat to escape, in contrast to the high temperatures reported by Lang (2014) under HT.

However, consistent with our hypothesis, we found a significant negative correlation of compression firmness with increasing average minimum RH (r^2^ = 0.96, p = 0.02). Cherry fruit firmness has been positively related to calcium concentration in the fruit, with sap flow providing the main pathway for calcium uptake and distribution in fruit trees (Zocchi and Mignani, 1995). Winkler et al. (2020) reported higher calcium concentrations in fruit grown at low RH (26.2%) in contrast to fruit grown at high RH levels (91.6%); similar results have also been reported in apple (Cline and Hanson, 1992; Tromp and Van Vuure, 1993). These findings support our results where a rapid decline in tree sap flow at RH ≥ 60% and higher minimum RH levels under rain covers limited tree sap flow, ultimately restricting sufficient transport of water and calcium into trees before movement to fruit for cell-wall development *via* the phloem. It is therefore thought that the lower average minimum RH levels recorded at location one throughout the season positively impacted fruit firmness characteristics where average temperatures were higher, but not excessive, throughout the season. However, location three had significantly higher sap flow levels in contrast to locations one and two yet recorded similar fruit compression firmness. This theory highlights that a number of factors are affecting fruit firmness and further research would be beneficial regarding tree calcium application, uptake and translocation to fruit under PCS.

#### DMC and TSS

In contradiction to our hypothesis, we found in this study that the correlation of both DMC and TSS with increasing daily temperature was positive. However this is consistent with the findings of Kafkaletou et al. (2015) who reported that moderate increases in average and maximum temperatures under PCS (0.9°C and 2°C respectively) were associated with increases in TSS in the cultivars ‘Adriana’ and ‘Noire de Meched’ compared to fruit from trees outside a PCS. The significant differences found in fruit DMC and TSS between fruit exposed to cooler average daily temperatures (lower GDD) in contrast to fruit exposed to higher average daily temperatures (higher GDD) are consistent with those reported by Lakatos et al. (2014). These authors found positive correlations in DMC and TSS content of sour cherry with the increased difference between daily maximum and minimum temperatures over a ten-year period in the cultivars “Debreceni bőtermő, Kántorjánosi, and Újfehértói fürtös”. Further support is provided by Blanke and Balmer (2008); Lang (2014), and Wallberg and Sagredo (2014), who all reported warmer temperatures under PCS leading to higher TSS levels in fruit.

## Conclusion

Whilst we found that time alone predicted approximately 90% of tree water use, our modelling approach that allowed for climate parameters to be held constant, demonstrated significant and distinct responses of sap flow to temperature, RH, solar radiation, and wind. However increases in temperature and reduced RH had the greatest impact on tree water uptake. Novel findings include that sap flow of sweet cherry continues to increase at high temperatures whilst high light induced a reduction in sap flows, giving a unique insight into the mechanism of control of sap flow. Further the substantial reduction in sap flows above the critical threshold of RH 60% may contribute to reductions in fruit firmness due to reductions in calcium uptake in fruit. The improvement in fruit quality characteristics (DMC, TSS and compression firmness) at higher elevations that were further from the boundary of the PCS, in contrast to fruit at low elevations closer to PCS boundaries, were associated with higher average temperature and lower minimum RH conditions developed under the PCS, demonstrating that the microclimate developed under the vented PCS was positive for fruit quality as opposed to unfavourable conditions that commonly develop under unvented HT PCS as reported in other studies. Overall, our findings on tree water uptake and fruit quality will better inform irrigation and fertigation practices at different locations under PCS to enable optimal crop management practices.

## Data availability statement

The raw data supporting the conclusions of this article will be made available by the authors, without undue reservation.

## Author contributions

Study conception and methodology were contributed to by DC, CS and SB. Material preparation and field data collections was performed by CS. Formal analysis was conducted by RC and interpretation of the analysis was undertaken by CS, DC and SB. The original draft of the manuscript was written by CS with data interpretation, writing, review and editing by DC and SB. All authors contributed to the article and approved the submitted version.

## Funding

This research was funded by Horticulture Innovation, grant number LP15007, as part of the Hort Frontiers strategic partnership initiative, with co-investment from The University of Tasmania and contributions from the Australian Government. This project was also supported by Fruit Growers Tasmania, Australia.

## Acknowledgments

We thank Hansen Orchards for their kind provision of the trial site and advice, Ian Goodwin for the provision of sap flow equipment and Ryan Warren for field and lab assistance.

## Conflict of interest

The authors declare that the research was conducted in the absence of any commercial or financial relationships that could be construed as a potential conflict of interest.

## Publisher’s note

All claims expressed in this article are solely those of the authors and do not necessarily represent those of their affiliated organizations, or those of the publisher, the editors and the reviewers. Any product that may be evaluated in this article, or claim that may be made by its manufacturer, is not guaranteed or endorsed by the publisher.

## Appendix

**A1:** Fitted spline curves of the residuals for each climatic predictor. To identify the best functional forms for the climatic predictors, separate GAM models were fitted for the residuals from each. The above GAM models used each of the covariates as a predictor, one at a time.

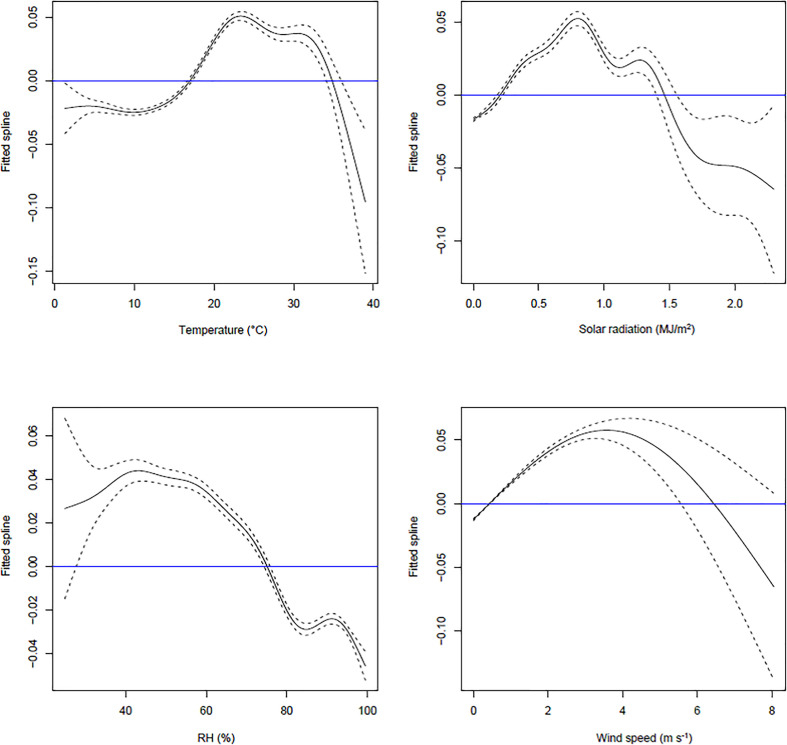

**A2:** Overall model illustrating the significance of each climatic predictor at each location under the PCS.

Parameter Estimate								
Parameter	Estimate	StandardError	DF	t Value	Pr > |t|	95% ConfidenceLimits		Gradient
M	28.5634	0.02985	165	956.98	<.0001	28.5044	28.6223	-0.00769
S	195.22	1.5862	165	123.08	<.0001	192.09	198.35	-0.00009
S2	0.006993	0.000056	165	124.96	<.0001	0.006883	0.007104	-3.68938
BT00	0.8471	0.02642	165	32.06	<.0001	0.7949	0.8992	-0.04109
BT1_1	1.7585	0.1508	165	11.66	<.0001	1.4607	2.0562	-0.01862
BT2_1	-1.3303	0.3788	165	-3.51	0.0006	-2.0782	-0.5824	-0.00451
C1_1	0.3093	0.01536	165	20.14	<.0001	0.2790	0.3397	-0.15434
C2_1	-0.1544	0.01025	165	-15.06	<.0001	-0.1746	-0.1342	-0.10398
R1_1	0.000423	0.000405	165	1.05	0.2972	-0.00038	0.001223	-0.32369
R2_1	-0.00987	0.000212	165	-46.45	<.0001	-0.01029	-0.00945	1.55572
ws1_1	-0.06397	0.004607	165	-13.89	<.0001	-0.07307	-0.05487	-0.12288
ws2_1	0.01285	0.001386	165	9.27	<.0001	0.01011	0.01558	-0.27452
BT1_3	1.3861	0.1520	165	9.12	<.0001	1.0861	1.6862	0.002949
BT1_2	0.4158	0.1602	165	2.60	0.0103	0.09953	0.7322	-0.02710
BT1_4	-0.2815	0.1559	165	-1.81	0.0728	-0.5892	0.02629	0.026090
BT2_3	-3.1242	0.4318	165	-7.23	<.0001	-3.9768	-2.2716	0.000383
BT2_2	-0.2333	0.4481	165	-0.52	0.6033	-1.1181	0.6514	-0.00578
BT2_4	2.3166	0.4601	165	5.03	<.0001	1.4081	3.2251	0.004543
C1_3	-0.1038	0.02033	165	-5.10	<.0001	-0.1439	-0.06363	-0.00960
C1_2	-0.1597	0.02032	165	-7.86	<.0001	-0.1998	-0.1195	-0.07669
C1_4	-0.07828	0.01914	165	-4.09	<.0001	-0.1161	-0.04048	-0.02359
C2_3	0.01664	0.01403	165	1.19	0.2372	-0.01106	0.04434	0.003778
C2_2	0.05353	0.01324	165	4.04	<.0001	0.02739	0.07967	-0.06169
C2_4	0.04786	0.01285	165	3.72	0.0003	0.02248	0.07324	-0.02315
R1_3	-0.00089	0.000519	165	-1.71	0.0883	-0.00191	0.000135	-0.37235
R1_2	-0.00359	0.000512	165	-7.02	<.0001	-0.00460	-0.00258	0.10328
R1_4	-0.00643	0.000507	165	-12.69	<.0001	-0.00743	-0.00543	-0.38554
R2_3	-0.00017	0.000167	165	-1.01	0.3123	-0.00050	0.000161	-5.02721
R2_2	-0.00084	0.000176	165	-4.75	<.0001	-0.00119	-0.00049	-19.2786
R2_4	-0.00097	0.000169	165	-5.71	<.0001	-0.00130	-0.00063	3.39630
ws1_3	0.06245	0.006345	165	9.84	<.0001	0.04992	0.07498	-0.00069
ws1_2	0.1050	0.006642	165	15.81	<.0001	0.09191	0.1181	-0.08803
ws1_4	0.07033	0.006863	165	10.25	<.0001	0.05678	0.08388	0.040992
ws2_3	-0.01129	0.001895	165	-5.96	<.0001	-0.01503	-0.00755	0.056054
ws2_2	-0.02049	0.002102	165	-9.75	<.0001	-0.02464	-0.01634	-0.19759
ws2_4	-0.01053	0.002267	165	-4.65	<.0001	-0.01501	-0.00605	-0.05071
sb	0.01715	0.001899	165	9.03	<.0001	0.01340	0.02090	0.095855
	Contrasts							
	Num Den							
Label	DF DF	F Value	Pr > F					
BT1	3 165	44.77	<.0001					
BT2	3 165	48.95	<.0001					
C1	3 165	21.40	<.0001					
C2	3 165	7.44	0.0001					
R1	3 165	65.66	<.0001					
R2	3 165	15.41	<.0001					
WS2	3 165	88.17	<.0001					
WS2	3 165	32.46	<.0001					

Each location is defined by a separate parameter in the above list for temperature, solar radiation, RH and wind speed. For example, location 1 is defined by BT1_1, BT2_1, C1_1, C2_1, R1_1, R2_1, WS1_1, and WS2_1. The overall F tests are shown under ‘Contrasts’. All terms are signficant, meaning that at least a pair of locations differs significantly
